# Evaluating professionalism in medical undergraduates using selected response questions: findings from an item response modelling study

**DOI:** 10.1186/1472-6920-11-43

**Published:** 2011-06-29

**Authors:** Paul A Tiffin, Gabrielle M Finn, John C McLachlan

**Affiliations:** 1 School for Medicine and Health, the Wolfson Research Institute, Durham University Queen's Campus, University Boulevard, Stockton-on-Tees, TS17 6BH, UK; 2 School for Medicine and Health, The Holliday Building, Durham University Queen's Campus, University Boulevard, Stockton-on-Tees, TS17 6BH, UK; 3 School for Medicine and Health, Durham University Queen's Campus, University Boulevard, Stockton-on-Tees, TS17 6BH, UK

## Abstract

**Background:**

Professionalism is a difficult construct to define in medical students but aspects of this concept may be important in predicting the risk of postgraduate misconduct. For this reason attempts are being made to evaluate medical students' professionalism. This study investigated the psychometric properties of Selected Response Questions (SRQs) relating to the theme of professional conduct and ethics comparing them with two sets of control items: those testing pure knowledge of *anatomy*, and; items evaluating the ability to integrate and apply knowledge ("*skills*"). The performance of students on the SRQs was also compared with two external measures estimating aspects of professionalism in students; peer ratings of professionalism and their Conscientiousness Index, an objective measure of behaviours at medical school.

**Methods:**

Item Response Theory (IRT) was used to analyse both question and student performance for SRQs relating to knowledge of professionalism, pure anatomy and skills. The relative difficulties, discrimination and 'guessabilities' of each theme of question were compared with each other using Analysis of Variance (ANOVA). Student performance on each topic was compared with the measures of conscientiousness and professionalism using parametric and non-parametric tests as appropriate. A *post-hoc *analysis of power for the IRT modelling was conducted using a Monte Carlo simulation.

**Results:**

*Professionalism *items were less difficult compared to the *anatomy *and *skills *SRQs, poorer at discriminating between candidates and more erratically answered when compared to *anatomy *questions. Moreover *professionalism *item performance was uncorrelated with the standardised Conscientiousness Index scores (rho = 0.009, p = 0.90). In contrast there were modest but significant correlations between standardised Conscientiousness Index scores and performance at *anatomy *items (rho = 0.20, p = 0.006) though not *skills *(rho = .11, p = .1). Likewise, students with high peer ratings for professionalism had superior performance on anatomy SRQs but not *professionalism *themed questions. A trend of borderline significance (p = .07) was observed for performance on *skills *SRQs and professionalism nomination status.

**Conclusions:**

SRQs related to professionalism are likely to have relatively poor psychometric properties and lack associations with other constructs associated with undergraduate professional behaviour. The findings suggest that such questions should not be included in undergraduate examinations and may raise issues with the introduction of Situational Judgement Tests into Foundation Years selection.

## Background

Promoting professionalism may be at once the most important and least successful aspect of medical training with well documented challenges in both defining [1] and assessing the construct [2]. Moreover, professionalism is a highly culture-bound construct and may vary according to the stage of educational development [3]. It is also unclear whether professionalism is a learned [4] or acquired characteristic. A recent study indicated that cases of completed disciplinary action were more likely to be men, to be of lower estimated social class, and to have had academic difficulties during their medical course, especially in the early years [5]. At least two of these three features are not attributable to the teaching of professionalism. If this proves indeed to be the case, a student could only be selected on the basis of professionalism, not taught it. In any event, the accuracy of evaluation of professionalism in medical students has implications for patient safety as well as individual development.

It is an assumption that professionalism has to be defined before it can be taught or measured. This may not be true: expert connoisseurship [6] can recognise situations which cannot be defined, just as a connoisseur may be able to recognise the quality of a new whisky without a checklist. Unsurprisingly there is no consensus on how to measure professionalism in undergraduates. Wilkinson has recently categorised five major themes in measuring professionalism [2]. These can be summarised as *adherence to ethical practice principles, effective interactions with patients and their significant others, effective interactions with other health professionals, reliability*, and *commitment to competence*. However, approaches to assessing professionalism have usually focussed on subjective decisions by those who have observed the candidate in action. Such measures are of low reliability, in that person-person interactions are strong, and the phenomenon of 'failure to fail' may apply, with assessors reluctant to dispense less than a pass grade [7]. This may be for a variety of reasons; the assessor may lack confidence in the assessment method; they may have formed a bond with the assessee; or they may just regard it as likely to cause too much trouble. Such assessments may have low validity, in that only the behaviour under test is scored, but attract a high economic cost given that such decisions are often made by senior clinicians. An attempt to mimic the reliability of Mini CEX has been pursued through the development of the Professionalism Mini-Examination PMEX [8]. This uses a scoring pad for observation of undergraduates in training. However, this instrument still suffers from the problems of a limited number of observations, 'failure to fail', and person-person interaction.

In the Durham University Medical Programme the measurement of diligence or conscientiousness has been explored as an index, or at least one component, of professionalism (the Conscientiousness Index) [9,10]. We have been able to demonstrate that there is a relationship between measures of conscientiousness in routine tasks and independent estimates of professionalism made independently by faculty and student peers. The measure also appears to have good reliability and Conscientiousness Index scores were found to be statistically significantly (p <.05) inversely correlated with the number of nominations for "least professional" individual by other students within their peer groups [9,10]. While this concurrent validity evidence is not of the same value as predictive validity evidence, it is none the less interesting as validated, objective, reliable and scalar information on professionalism in undergraduate medical students. This gives us an opportunity to explore relationships between the Conscientiousness Index and other potential measures of professionalism which may be used as predictors of future performance.

One US-based study reported a negative association between assessment performance during internships and the likelihood of referral for disciplinary action in later medical careers [11]. Moreover, the authors reported a positive relationship between evidence of poor professionalism ratings during internships and the likelihood of referral for disciplinary action in later medical careers. Both findings could be explained by conscientiousness acting as a mediator between assessment performance and professionalism ratings. This issue is particularly relevant at present, since it is proposed that Situational Judgement Tests (SJTs) will be used for the high stakes selection of candidates for Foundation places in the UK from 2012. These SJTs themselves take the form of Selected Response Questions (SRQs) whereby a candidate is offered a short written vignette concerning a complex work situation and selects one or more of the most appropriate professional responses from a list of responses [12]. There is evidence that these are positive predictors for workplace performance [13]. However, they have not been tested with regard to undergraduate performance. In addition, SJTs and knowledge-based tests are currently used as measures of performance with regard to professionalism in some undergraduate curricula. The argument could be made that, although knowledge of the ethical course of action is not evidence of an intention to act ethically, it is an essential prerequisite. We have therefore analysed undergraduate student performance on SRQs in comparison with their conscientiousness and peer ratings of professionalism. Our aim was to evaluate whether there was any evidence to support the use of SRQs when evaluating professionalism. The primary objective was to compare both item and student person performance on SRQs concerned with professional behaviour with two other types of control question. Thus, our hypothesis was that ratings of professionalism and conscientiousness would be more strongly associated with performance on SRQs probing knowledge of professional conduct compared to other types of item.

## Methods

### Study Design

A cross-sectional survey design was utilised in order to examine the relationship between the variables under study. Data from two consecutive cohorts of medical undergraduates during their first two years at medical school were utilised.

### Data collection

There were 96 students in the first cohort and 98 in the second. Examination results were available from four examinations for the first cohort and three examinations for the second. The SRQ-based examinations conducted in the first two years consist of multiple choice question (MCQ) items, where a single best answer was selected from a choice of five responses, and Extended Matching Questions (EMQs). In the case of EMQs each item has a themed list of possible responses and multiple questions linked to this with the candidate aiming to match a response to each question. At Durham University the examinations conducted in years I and II cover a wide range of topics including immunology, microbiology, anatomy (pure and applied), medical ethics and physiology. In turn, items are allocated to three main categories: 'Knowledge and Critical Thinking'; 'Skills'; and 'Professional Behaviours'. The Professional Behaviours domain includes both reflective writing and understanding of how to behave professionally. Within the four examinations the first cohort answered 14 MCQs and 25 EMQs (i.e. 5 sets of response lists) on *professionalism*. The second cohort answered eight MCQs and 20 EMQs (i.e. 4 response lists) on this domain. An example of a professionalism MCQ item would be; "*You are on your Community Placement which offers bereavement counselling. In one session the placement worker, who you are shadowing, deals harshly with a crying client. This has never happened before. Do you:*

a) challenge the placement worker in front of the client?

b) pretend it didn't happen and say/do nothing?

c) take over the counselling session yourself ?

d) confront the placement worker afterwards in private?

e) report the placement worker to his/her superior?"

In order to explore the properties of the *professionalism *items they were compared to the responses to questions relating to "pure" (as opposed to applied) knowledge of anatomy. This theme was selected to serve as a control set of items as it was hypothesised that acquisition of anatomical knowledge was more likely to require conscientious study than knowledge of professionalism. The first cohort answered 22 MCQs and 55 EMQs and the second cohort answered seven MCQs and 25 EMQs on anatomy. A third set of SRQs, taken from the 'Skills' category of question was also included as an alternative comparison group. These items were designed to test the skill of drawing on knowledge (sometimes from different topics) and applying the information to clinical problems. An example of a *skills *themed SRQ would be; "*Radiological imaging is commonly used in the investigation of the hepatobiliary and GI tract. Which of the following statements is true when a clinician is considering what type of image to request?*

a) The skill of the operator is paramount in obtaining a plain abdominal film

b) Fluoroscopy cannot demonstrate oesophageal motility

c) A double contrast barium enema will rarely visualise the caecum

d) Magnetic resonance imaging exposes the patient to considerably less ionising radiation than computerised tomography

e) Endoscopic retrograde pancreatography is very useful to assess pancreatic function"

Responses from the first cohort to 24 MCQs and 29 EMQs from the *skills *category were analysed. For the second cohort 16 MCQs and 5 EMQs relating to *skills *were utilised. The responses to these items were analysed using a Rasch analysis (see below) in order to generate an interval metric of estimated student ability in relation to *knowledge of professionalism, anatomy *and *skills*.

Data relating to the Conscientiousness Index was also available for each student as a percentage of the total "conscientiousness points" available. This measure relies on objective information such as attendance at teaching sessions and compliance with administrative tasks such as submission of immunisation documentation [10]. In order to compare the two cohorts accurately the Conscientiousness Index percentages were converted to standardised z scores. In addition, information was available relating to peer nominations for professionalism. This approach has been previously shown to detect "extremes" and those students who have received a high number of nominations for being perceived as "least professional" had, on average, lower Conscientiousness Index scores [9]. In the first cohort peer nominations were conducted within the peer group. In order to increase participation, for the subsequent cohort, peer assessment was conducted within tutor groups. This change was made because students had reported they felt it was easier to make accurate nominations within a tutor group where there was more familiarity with peers, rather than within a year group. For both year groups nominations were converted into an aggregate score of professionalism by subtracting nomination for *least professional *from those for *most professional*. Cut-offs were generated in order to identify the top 10% and bottom 10% of aggregate scores within each year group. Thus students were categorised as having peer professionalism ratings that were high, low or neither.

### Item response modelling

Item response modelling and theory (IRT) is based on the modified factor analysis of binary and categorical data. Within the family of IRT models Rasch analysis was originally developed for the exploration of dichotomous responses to test items [14]. Rasch analysis can be used to create interval metrics of both item difficulty and respondent ability from ordinal (ordered categorical) or binary (dichotomous) response data. The Rasch model assumes that all items are identical in terms of their ability to discriminate between respondents according to ability (i.e. equality of item factor loadings in classical factor analytic terms). Nevertheless, Rasch software is able to provide simulated estimates of other parameters aside from difficulty and ability such as the degree of discrimination an item provides in determining the level of the underlying trait in a respondent. In addition, an estimated value for a lower asymptote is provided which represents an index of "guessing". Normally these latter values are estimated using the less constrained two and three parameter (2-PL, 3-PL) logistic models rather than the Rasch model. The WINSTEPS programme is able to provide indices of these parameters which are purported to be as accurate as those provided by less constrained models [15-17]. In a Rasch analysis reliability can be appraised in a number of ways; the person reliability coefficient relates to the replicability of the ranking of abilities while the person separation index represents the signal to noise ratio and estimates the ability of a test to reliably differentiate different levels of ability within a cohort [18]. A description of IRT and its potential application in a medical education setting has been previously published [19].

The Rasch analysis was conducted in two ways. Firstly, to construct interval measures of performance at each type of question, the items of each type were pooled and analysed by cohort. For example, for performance on *professionalism *items included in the first cohort's examinations all responses to items relating to this theme were pooled across exams and Rasch analysed as a batch. Estimates of ability were derived for both MCQ and EMQ format items in order to evaluate whether the two types of items should be combined. Reliable test-equating between examinations sat by different cohorts was not possible as there were no common items included. For this reason ability estimates on the three domains (*skills, anatomy *and *professionalism*) were standardised as z scores for each cohort. Secondly, the relative item characteristics for each theme (*skills, anatomy *and *professionalism*) were compared by performing a Rasch analysis separately for each exam.

The Rasch model assumes local independence (i.e. there should be no correlation between responses once the "Rasch dimension" has been controlled for). If this assumption is violated then values such as ability and person separation estimates may be overestimated. In the case of EMQs the item responses are related to the same stem. Thus, there was a risk that this assumption of local independence would not hold either because the response related to a particular area of specialised knowledge or the stem question posed was asked in a particular way (i.e. a method effect). For this reason we examined the data for evidence of systematic non-independence in the responses as evidenced by correlated residuals between responses to EMQs relating to the same stem. There were surprisingly few relatively large (i.e. > 0.3) correlated residuals observed, with most sets of items having one or no pairs of correlated residuals present (in some cases these were not even between items relating to the same stem). The effect of such local dependency was evaluated using the method recommended by Linacre [16]. Firstly "testlets" of locally dependent items were produced by summing their responses. The model was then re-estimated using the partial credit Rasch model (which accommodates more than two response categories). The old and new person ability estimates recovered from the model were then cross-plotted with the original values obtained and examined for evidence of change. No obvious changes in the estimates were noted, the only exception being the anatomy SRQs completed by the first cohort. In this case eight of the 33 items were found to have correlated residuals (seven of which were related to the same stem). When testlets of items with correlated residuals were constructed and entered into the model around 24% of the anatomy ability estimates (relating to 23 students) for that cohort markedly changed (i.e. departed from the diagonal of the cross-plot). For this reason performance on the *anatomy *SRQs for the first cohort was estimated using this method. Likewise, the person separation index for anatomy was calculated on the basis of this analysis using testlets.

Of the 1,064 items evaluated from the seven exams ten had not been scored due to problems with wording/ambiguity that were discovered after administration. A further 21 items were answered correctly by every student and therefore did not provide any information. When comparing the *professionalism *items with those of other themes such items that had been answered correctly in all cases (i.e. those where the difficulty could not be calibrated) were included when analysing the comparative facility of the questions. In these cases such items were assumed to be very easy and assigned an arbitrary difficulty of -5 logits to reflect this. The value of -5 was selected as it was consistent with the lowest difficulty scores for those items where information was available. Item difficulty estimates were normally distributed (when the items where difficulty had been fixed at -5 logits were excluded) and therefore Analysis of variance (ANOVA) was used to assess for intergroup differences. Discrimination estimates were significantly skew and therefore intergroup differences were compared using a Kruskal-Wallis test.

Power issues in Rasch analysis are a matter for debate with some authors suggesting that around 200 respondents are required to accurately estimate item difficulty whilst others suggest as few as 30 participants may be required in well-targeted tests (i.e. those where difficulty is well matched to ability) [20-22]. For this reason a post-hoc power exploration was performed using a Monte Carlo simulation study [23]. This was carried out in two stages according to the method described by Muthén and Muthén [24] as implemented in Mplus version 5.21 [25]. The simulation used responses from the smaller first cohort and was conducted over 10,000 iterations. The results were examined for evidence of bias in the replicated item difficulty values [23].

For normally distributed variables pairwise correlations and ANOVA were performed in STATA version 10 [26]. Where the variable was observed to be non-normally distributed according to a significance test [27] then an appropriate non-parametric comparison was performed.

### Ethical Approval

The SRQ data utilised by this study was routinely gathered for assessment and course monitoring purposes. Anonymity was maintained for all students during the analysis process by use of a unique identifier code. Students were advised that such data was being collected and could be used in non-identifiable form. Ethical suitability of these studies for publication was confirmed in writing by the Chair of the School's Ethics Committee. Other data used in the present analysis was collected as part of research that had been given ethical approval by the Durham University School for Health Research Ethics Committee. It has previously been argued that data collected for routine assessment purposes may be subsequently used for research purposes as long as the data is anonymised, and no harms can result from its use [28].

## Results

### Exam Item Characteristics

Where a trait or ability conforms to the assumption of unidimensionality made by the Rasch model there should be relatively little correlation between responses once the effect of the underlying dimension has been removed. In the present study the "Rasch factor analysis" findings generally supported this assumption in that the contrasts within the residuals from a Rasch Factor Analysis consistently explained less than approximately 5% of the unexplained variance in item responses [16]. However, the Rasch factor analysis for the *skills *items completed by the second cohort suggested the presence of at least a second dimension indicated by the first contrast in the residuals explaining 7.5% of the variance. The item characteristics, as estimated by the Rasch analysis, are depicted in Table 1.

**Table 1:** Item characteristics relating to the themes of professionalism, anatomy or skills from the seven exams taken by the two cohorts attending years I and II of medical school at Durham University

Items	Difficulty (sd) Logits	Discrimination (sd)	Z Infit (sd)	Z Outfit (sd)	Guessing Index (sd)
Anatomy MCQs	.54 (1.2)	1.08 (.3)	-.27 (.8)	-.30 (.9)	.02 (.1)
Anatomy EMQs	-.47 (1.6)	1.07 (.2)	-.17 (.6)	-.35 (.7)	.06 (.2)
Anatomy Combined	-.16 (1.6)	1.08 (.2)	-0.2 (.7)	-.33 (.8)	.05 (.2)
Skills MCQs	-.57 (1.9)	.91 (.3)	.27 (.8)	.38 (1.0)	.11 (.3)
Skills EMQs	-.09 (1.7)	.92 (.2)	.29 (.6)	.33 (.8)	.05 (.2)
Skills Combined	-.35 (1.8)	.92 (.3)	.28 (.7)	.35 (.9)	.08 (.2)
Prof. MCQs	-.38 (2.0)	.81 (.5)	.60 (1.1)	.82 (1.1)	.19 (.3)
Prof. EMQs	-1.47(1.8)	.94 (.2)	.29 (.5)	.34 (.7)	.07 (.2)
Prof. Combined	-1.11( 1.9)	.90 (.3)	.39 (.8)	.50 (.9)	.11 (.3)

The estimates of relative item difficulty, discrimination, standardised "infit"/"outfit" and a "guessing index" are depicted with their respective standard deviations.

Candidates performed significantly better on *Professionalism *items compared to *anatomy *(F = 13.44, p < 0.001) and *skills *questions (F = 6.04, p = .02). In addition the estimates of the *professionalism *item discrimination parameters were significantly lower compared to those for *anatomy *(F = 19.55, p = < 0.001) but not *skills *items (F = .14, p = .7). This implies that the *professionalism *items were easier compared to the other two item types and poorer at discriminating candidates of differing abilities compared to the *anatomy *items. In terms of the fit of item responses to the Rasch model, responses to *anatomy *items were mildly skew towards overfitting the model according to 'infit' (information weighted) indices: the average z score for infit for *anatomy *items was -.20 reflecting a tendency to less variation in responses than the Rasch model would have predicted. In contrast, the *professionalism *items were skew towards underfit with a mean z score of .39. This reflected a trend to a slightly more erratic response pattern than might be expected under the assumptions of the Rasch model. *Skills *items had fit indices intermediate between these two former themes. Thus, *anatomy *item performance appeared to be more predictable than the response patterns observed for the *professionalism *items.

Person reliability indices were relatively high for estimation of ability at *anatomy *items: for the first cohort the person reliability index .82 and the person separation value was 2.15 (for the second cohort these values were .73 and 1.63 respectively). In contrast the person reliability indices for *professionalism *and *skills *items were much lower: for *professionalism*, person reliability was 0.32 and separation was 0.69 for the first cohort. For the second cohort these values were .43 and .87 respectively. For *skills *items person reliability was 0.42 and separation was 0.85 for the first cohort. For the second cohort these values were .42 and .85 respectively. This implies that both *professionalism *and *skills *items have a limited ability to discriminate between high and low performers on these measures.

### Relationship between ability estimates and conscientiousness/professionalism

The performance estimates derived from EMQs and MCQs were highly correlated. For example, ability at *anatomy *items as evaluated by performance at both EMQs and MCQs correlated highly with ability solely judged by relevant MCQs (r = 0.80) and EMQs (r = 0.94). For this reason the performance estimates utilised were those derived from analysis of both SRQ formats for the relevant items. Performance estimates for the SRQs were normally distributed. However, Conscientiousness Index scores were significantly skew, therefore Spearman's rank correlation test was used when comparing this variable with others. Performance on *professionalism *items was not significantly correlated with *anatomy *performance (r = .12, p = .1). In contrast, *professionalism *and *skills *performance was modestly correlated (r = .27, p <.001) as was ability at *anatomy *and *skills *items (r = .35, p <.001).

*Professionalism *item performance was uncorrelated with the standardised Conscientiousness Index scores (rho = 0.009, p = 0.90). A slight non-significant trend was noted for performance on *skills *items (rho = 0.11, p = 0.1) and Conscientiousness Index scores. In contrast there were modest but significant correlations between standardised Conscientiousness Index and performance on *anatomy *items (rho = 0.20, p = 0.006). Analysis of variance was also used to test for standardised performance on the SRQs and Conscientiousness Index according to peer professionalism aggregate score category (high professionalism, low professionalism or neither). The results are depicted in Table 2, highlighting a number of intergroup performance differences, though notably not on the *professionalism *SRQs, where differences did not reach statistical significance (p ≥ .1 in all cases).

**Table 2:** Standardised performance on Conscientiousness Index z scores and the three groups of Selected Response Questions (SRQs- logit z scores) according to peer rating category for students in both cohorts (N = 194)

Peer Ratings of Professionalism	Conscientious. Index z scores*	*Anatomy *SRQ Performance Mean (SD)	*Skills *SRQ Performance Mean (SD)	*Professionalism *SRQ Performance
High (N = 13)	.83 (.7)**	.75(1.1)*	.21(.9)^§^	.33(1.1)
Neither (N = 163)	.01(1.0)**	-.06(.9)	.02(1.0)	.02(1.0)
Low (N = 16)	-.74(1.1)**	.33 (1.1)	-.40(.9)	-.34(1.0)

** All intergroup differences significant at the p <.01 level

* Intergroup difference between "High" and "Neither" group significant at the p <.01 level

^§ ^Intergroup difference between "high" and "low" group of borderline significance at p = .07

### Findings from the Monte Carlo simulations

The Monte Carlo simulation suggested that, in general, the difficulty estimates were well replicated for both the *anatomy *and the *professionalism *items with bias of around 1-2%, even when using the smaller cohort of 98 students. However this was not true for a number of very easy "mistargeted" items with difficulty values of -3.0 logits or less (as scaled according to person ability) where bias was 8.6 to 110%. For the overall *professionalism *items the average bias between the actual population and simulated values was 10.9%. However when the seven very easy items with were excluded an average bias of 1.2% was observed. Likewise, the simulated and actual estimates of item difficulty for the *anatomy *items were generally between 1-5% with the exception of ten very easy items of difficulty -3 logits or less. When these were excluded the average bias in the estimates was 1.9%. These results implied that, with the exception of this small number of "mistargeted" questions, the study was adequately powered to estimate the item characteristics accurately.

## Discussion

According to the IRT-based analysis, the psychometric properties of the *professionalism *SRQs were inferior to those of items relating to the testing of knowledge of anatomy. In particular *professionalism *items were relatively poor at discriminating between candidates. This is especially highlighted by the low person separation indices observed for these items; in order to reliably discriminate between two groups of candidates a person separation index of more than two would be required. In the case of the *professionalism *items these values were much less than one. The relationship between Conscientiousness Index scores, professionalism peer nominations and performance at *anatomy *SRQs were modest but statistically significant. However, no such relationships were observed between these former measures and performance at *professionalism *items. The third set of *skills *items, relating to the application of knowledge were observed to have psychometric properties somewhat intermediate between those of the *professionalism *and *anatomy *items. Although there were no statistically significant associations between performance on *skills *items and the ratings of conscientiousness and professionalism there was at least the suggestion of a trend. As with the *professionalism *items, the person separation indices for the *skills *items were relatively low. Taken together the characteristics of the three types of item may imply that the testing of applied, as opposed to pure, knowledge is generally less reliable using the SRQ format. This possibility may at least partly explain the poor psychometric properties of the *professionalism *items, which suggest that SRQs may not be an appropriate measure or predicator of professionalism, at least for undergraduate medical students.

Whilst some clinical exposure occurs during the first two years of Durham University Medical School training, knowledge based-performance is still the main focus of study. Therefore, conscientious study may be more closely allied to peer perceptions of professionalism than in later stages of medical training, where more patient and staff interactions are observed amongst peers. It could be argued that performance on anatomy items most closely reflects this aspect of professionalism, given that without conscientious study it is difficult to perform well on this topic. However, the converse was not true in that those that peers perceived as least professional did not demonstrate a poorer performance on any area assessed by SRQs. This suggests that medical students may be relatively accurate at perceiving high but not low levels of conscientiousness, in contrast to previous findings where Conscientiousness Index was associated with low but not high ratings of professionalism. This apparent anomaly could be due to the wider definition of Conscientiousness Index, which encapsulates a range of information on behaviour, in contrast to anatomy performance which is restricted in scope. Thus these two correlates of conscientiousness may be related to professionalism in subtly different ways.

It is also necessary to explain why the present findings seem to be at odds with those reported by Patterson et al; that SJTs predict workplace performance by GP trainees [13]. There are two possible explanations. Firstly, professionalism may be developmental in nature, and perhaps early undergraduate medical students do not respond appropriately to SJTs because they have not yet developed appropriate situational judgement. The other, more encouraging version, is that SJTs measure aspects of professionalism different from those measured by the Conscientiousness Index. The strongest association we have found between the Conscientiousness Index and professionalism suggests that conscientiousness accounts for 25% of the variance in professionalism. While this is the largest single component that has been identified, at least to our knowledge, it leaves room for other, unknown, components to play significant roles, and there is no reason to believe that these co-vary with conscientiousness. The other four members of Psychology's 'Big Five' (extroversion, neuroticism, agreeableness and openness to experience [29]) would be obvious candidates. Equally, Wilkinson identifies five clusters of measures of professionalism, one of which clearly correlates with conscientiousness, and the other four may well represent different aspects of professionalism [2]. In addition, it is possible that increased patient exposure in later training years may increase students understanding of the correct response in clinical situations and lead to more consistent responses to items related to professional behaviour.

Rasch analysis has previously shown to be a useful approach when exploring the psychometric properties of medical undergraduate exam SRQs [30]. Although not the focus of the present study, the findings from the present Rasch analysis of the exam items also suggest that SRQ format (e.g. EMQ versus MCQ) may influence their characteristics in a topic specific way. This observation merits further research. More importantly, the findings from the present study should raise some concerns regarding the use of SJTs for selection to Foundation, as proposed by the Medical Schools Council. As the candidates for these latter high-stakes assessments fall between undergraduate and postgraduate supporting evidence regarding the properties of these tests in populations at that stage of professional development is urgently required. If these tests do not perform adequately it may result in strong candidates failing to obtain one of their preferred foundation year posts, or in the worst case scenario, any post at all.

### Strengths and limitations

This is the first published study to combine two distinct indices of professionalism with SRQ performance, using an IRT approach. The application of IRT allowed an interval metric of ability to be constructed from the exam question responses. Moreover, the psychometric properties of the items could be explored more fully than classical test-theory would normally allow. Ideally the ratings of professionalism in the two year groups would have been both derived from tutor group ratings and therefore some caution must be exercised in interpreting the professionalism nominations. One of the strengths of IRT is the ability to derive relatively distribution free measures of performance. It would therefore have been desirable to use test-equating via shared items to link absolute-SRQ ability across year groups rather than standardised Rasch scores, although the lack of shared questions precluded this.

The SRQ response data utilised in this study did not include sociodemographic variables, such as gender and ethnicity. Thus, it was not possible to assess the response data for the presence of differential item functioning (DIF- response bias not due to underlying ability) according to such candidate characteristics. This may be an important area of future research.

The Monte Carlo simulation suggested that the item difficulty estimates were precise and reliable in the majority of cases. However, item discrimination and guessing parameters should ideally be evaluated via a full two-parameter logistic model, rather than estimated using the more constrained Rasch model. Thus, the application of IRT, whilst possible with a relatively small number of respondents, is more suited to larger population samples.

## Conclusion

The findings of this study imply that SRQs relating to the theme of professional behaviour are likely to have poor psychometric properties and suggests that such questions should not be routinely included in medical school exams. Further work could explore whether these results generalise to the use of SJTs in later stages of medical training. Efforts should be directed at developing reliable and valid estimates of professionalism combining multiple data sources.

## Competing interests

The authors declare that they have no competing interests.

## Authors' contributions

PAT led on conception, design, statistical analysis and interpretation of data and is the guarantor of the paper. JMcL contributed to drafting, revising the article and critically appraising the content. GMF was responsible for conception, design and execution of the study component concerned with conscientiousness index and peer ratings. In addition GMF has contributed to the drafting of the article. All authors (PAT, JMcL and GMF) have approved the final version of the article submitted.

## Authors' information

PAT is a Clinical Senior Lecturer in the Psychiatry of Adolescence and has a Medical Doctorate (MD) in the area of psychometrics, having an interest in statistical modelling approaches in the behavioural sciences. GMF is a Lecturer in anatomy and has completed a PhD based on the assessment of conscientiousness and professionalism in medical undergraduates. JCMcL is Professor of Medical Education and has an interest in the assessment of professionalism in medical settings.

## Pre-publication history

The pre-publication history for this paper can be accessed here:

http://www.biomedcentral.com/1472-6920/11/43/prepub

## References

[B1] Van De Camp K Vernooij-Dassen MJFJ Grol RPTM Bottema BJAM How to conceptualize professionalism: a qualitative study Medical Teacher 2004 26 696 702 10.1080/01421590400019518 15763872

[B2] Wilkinson TJ Wade WB Knock LD A Blueprint to Assess Professionalism: Results of a Systematic Review Academic Medicine 2009 84 551 558 10.1097/ACM.0b013e31819fbaa2 19704185

[B3] Wagner P Hendrich J Moseley G Hudson V Defining medical professionalism: a qualitative study Medical Education 2007 41 288 294 10.1111/j.1365-2929.2006.02695.x 17316214

[B4] Hilton SR Slotnick HB Proto-professionalism: how professionalisation occurs across the continuum of medical education Medical Education 2005 39 58 65 10.1111/j.1365-2929.2004.02033.x 15612901

[B5] Yates J James D Risk factors at medical school for subsequent professional misconduct: multicentre retrospective case-control study BMJ 2010 340 c2040 10.1136/bmj.c2040 20423965 PMC3191727

[B6] Bleakley A Farrow R Gould G Marshall R Making sense of clinical reasoning: judgement and the evidence of the senses Medical Education 2003 37 544 552 10.1046/j.1365-2923.2003.01542.x 12787378

[B7] Cleland JA Knight LV Rees CE Tracey S Bond CM Is it me or is it them? Factors that influence the passing of underperforming students Medical Education 2008 42 800 809 10.1111/j.1365-2923.2008.03113.x 18715477

[B8] Cruess R McIlroy JH Cruess S Ginsburg S Steinert Y The Professionalism Mini-Evaluation Exercise: A Preliminary Investigation Academic Medicine 2006 81 S74 S78 10.1097/00001888-200610001-00019 17001141

[B9] Finn G Sawdon M Clipsham L McLachlan J Peer estimation of lack of professionalism correlates with low Conscientiousness Index scores Medical Education 2009 43 960 7 10.1111/j.1365-2923.2009.03453.x 19769645

[B10] McLachlan J Measuring conscientiousness and professionalism in undergraduate medical students The Clinical Teacher 2010 7 37 40 10.1111/j.1743-498X.2009.00338.x 21134141

[B11] Papadakis MA Teherani A Banach MA Knettler TR Rattner SL Stern DT Disciplinary action by medical boards and prior behavior in medical school New England Journal of Medicine 2005 353 2673 82 10.1056/NEJMsa052596 16371633

[B12] Medical Schools Council Improving Selection to the Foundation Programme 2010 London: MSC

[B13] Patterson F Baron H Carr V Plint S Lane P Evaluation of three short-listing methodologies for selection into postgraduate training in general practice Medical Education 2009 43 50 7 10.1111/j.1365-2923.2008.03238.x 19140997

[B14] Rasch G Probabilistic models for some intelligence and attainment tests 1960 Copenhagen. Danish Institute for Educational Research

[B15] WINSTEPS^® ^Rasch measurement computer program [program]. 3.67.0 version 2009 Beaverton, Oregon: Winsteps

[B16] Linacre JM A User's Guide to WINSTEPS: Program Manual 3.69.1 2010

[B17] Linacre JM Detecting Multidimensionality: Which residual data-type works best? Journal of Outcome Measurement 1998 2 266 283 9711024

[B18] Wright BD Masters GN Rating Scale Analysis 1982 Chicago. MESA Press

[B19] Downing SM Item response theory: applications of modern test theory in medical education Medical Education 2003 37 739 745 10.1046/j.1365-2923.2003.01587.x 12945568

[B20] Baur T Lukes D An Evaluation of the IRT Models through Monte Carlo Simulation UW-L Journal of Undergraduate Research 2009 XII 1 7

[B21] Goldman SH Raju NS Recovery of One- and Two-Parameter Logistic Item Parameters: An Empirical Study Educational and Psychological Measurement 1986 46 11 21 10.1177/0013164486461002

[B22] Linacre JM Sample Size and Item Calibration Stability Rasch Measurement Transactions 1994 7 328

[B23] Muthén LK Muthén B How to use a Monte Carlo study to decide on sample size and determine power Structural Equation Modeling 2002 4 599 620

[B24] Muthén LK Muthén BO Mplus User's Guide 2007 5 Los Angeles, CA. Muthén and Muthén

[B25] Mplus [program]. 5.21 version 2009 Los Angeles, LA: Muthén & Muthén

[B26] Intercooled Stata for Windows [program]. 10.0 version 2007 College Station: Stata Corporation

[B27] D'Agostino RB Balanger A D'Agostino RBJ A suggestion for using powerful and informative tests of normality American Statistician 1990 44 316 321 10.2307/2684359

[B28] McLachlan JC McHarg J Ethical permission for the publication of routinely collected data Medical Education 2005 39 944 948 10.1111/j.1365-2929.2005.02223.x 16150035

[B29] Costa PT Macrae RR The NEO PI-R Professional Manual 1992 Odessa, FL. Psychological Assessment Resources Inc

[B30] Bhakta B Tennant A Horton M Lawton G Andrich D Using item response theory to explore the psychometric properties of extended matching questions examination in undergraduate medical education BMC Medical Education 2005 5 9 10.1186/1472-6920-5-9 15752421 PMC555598

